# Ischemic stroke-related gene expression profiles across species: a meta-analysis

**DOI:** 10.1186/s12950-023-00346-x

**Published:** 2023-06-19

**Authors:** Ruslan Rust

**Affiliations:** 1 Institute for Regenerative Medicine (IREM), University of Zurich, Campus Schlieren Wagistrasse 12, Schlieren, Zurich, 8952 Switzerland; 2 Neuroscience Center Zurich, University of Zurich and ETH Zurich, Zurich, Switzerland

**Keywords:** Meta analysis, Ischemia, Hypoxia, Stroke, Preclinical research, Transcriptomics, Gene expression, Gene ontology, Animal models

## Abstract

Stroke patients are often left with permanent disabilities with no regenerative treatment options. Unbiased RNA sequencing studies decoding the transcriptional signature of stroked tissue hold promise to identify new potential targets and pathways directed to improve treatment for stroke patients. Here, gene expression profiles of stroked tissue across different time points, species, and stroke models were compared using NCBI GEO database. In total, 34 datasets from mice, rats, humans, and primates were included, exploring gene expression differences in healthy and stroked brain tissue. Distinct changes in gene expression and pathway enrichment revealed the heterogenicity of the stroke pathology in stroke-related pathways e.g., inflammatory responses, vascular repair, remodelling and cell proliferation and adhesion but also in diverse general, stroke-unrelated pathways that have to be carefully considered when evaluating new promising therapeutic targets.

**Supplementary Information:**

The online version contains supplementary material available at 10.1186/s12950-023-00346-x.

## Background

Stroke is a major cause for disability and death affecting one in four people during their lifetime [1]. Stroke survivors have only limited therapeutic options and are often left with considerable disabilities [2, 3]. The development of new therapeutics for stroke is not trivial as the ischemic cascade involves many pathological processes such as cellular excitotoxicity, oxidative stress, inflammation, blood-brain barrier disruption and scarring [4]. Therefore, unbiased screening studies decoding the transcriptional response of stroked tissue are important to identify new potential targets for therapy [5, 6]. Since human stroke brain tissue is only of limited suitability for the analysis (i.e., it can only be accessed in lethal strokes and the processing time may lead to poor RNA quality), the majority of transcriptional profiling of stroked tissue was performed in rodents. Although animal models are designed to generate reproducible infarcts in highly controlled conditions, there is recognized heterogeneity among stroke models and species [7]. The most commonly used stroke model is the middle cerebral artery occlusion (MCAo) model; in this model the most commonly affected artery in human stroke is surgically obscured. The occlusion can either be transient to produce reperfusion after 30–120 min (tMCAo) or permanent (pMCAo) [8, 9]. Alternatively, the photothrombotic stroke model is popular for permanent ischemia in defined brain regions that lead to long-term functional deficits [9–11].

In this study, the gene expression omnibus (GEO) RNA sequencing data were obtained from stroke tissue across different stroke models, sex, time points, and species. The gene expression profiles were integrated to identify and compare common and differentially expressed genes (DEG) and enriched pathways in the individual groups.

## Methods

### Meta analysis procedure

data search from GEO microarray data repositories were searched in November 2022. The search terms were “stroke”, “ischemia”, “tMCAo”, “pMCAo”, “MCAo”, “photothrombotic stroke”. Selected organisms were “homo sapiens”, “macaca”, “mus musculus” and “rattus norvegicus”. Datasets were excluded that did not include brain tissue samples (Suppl. Figure 3). (Most human studies 49/51 had to be excluded because they performed RNAseq with blood samples). Further studies were excluded with missing information, duplicates, pooled samples, poor quality controls, and no clear separation between stroked and non-stroked groups. No separation between stroke and non-stroke groups was considered to be a result of either a failed stroke induction or incorrect allocation of datasets, leading to the exclusion of these studies. An overview of the used datasets can be found in Suppl. Table 1. Significant genes for each group were identified using R Studio RankProd [12]. All datasets were annotated and converted uniformly using genome wide annotation resources.

### Study selection

The search initially retrieved 338 articles, of which 34 met the inclusion criteria.

### Data processing and annotation

First, all data frames were pre-processed and normalized using the trimmed mean of M-values TMM in edgeR to account for differences in library size, sequencing depth, and gene length and reduce technical variability. Gene annotation and identifiers across species was performed using biomaRt mapIDs package. All molecular identifiers were converted in gene symbols and capitalized (to account for different species). Comparison of different species, time points and stroke model were performed by calculating the changes between stroked and the corresponding control transcriptome using the edgeR package and then comparing the overlap of up- and downregulated genes via Venn diagrams.

### Venn Diagram

Overlap between upregulated and downregulated genes were visualized using venn.diagram function in R. Differentially expressed genes with fold change +/- 0.5 were compared from different species, time points and stroke models.

### Functional enrichment analysis of DEGs

Analysis of functional enrichment analysis for DEGs was performed using EdgeR [13] and clusterProfiler 4.0 using the function gseGO [14] in RStudio.

## Results

### Gene expression profiles of stroked brain tissue across sex, species, stroke model, and time

**Fig. 1:**
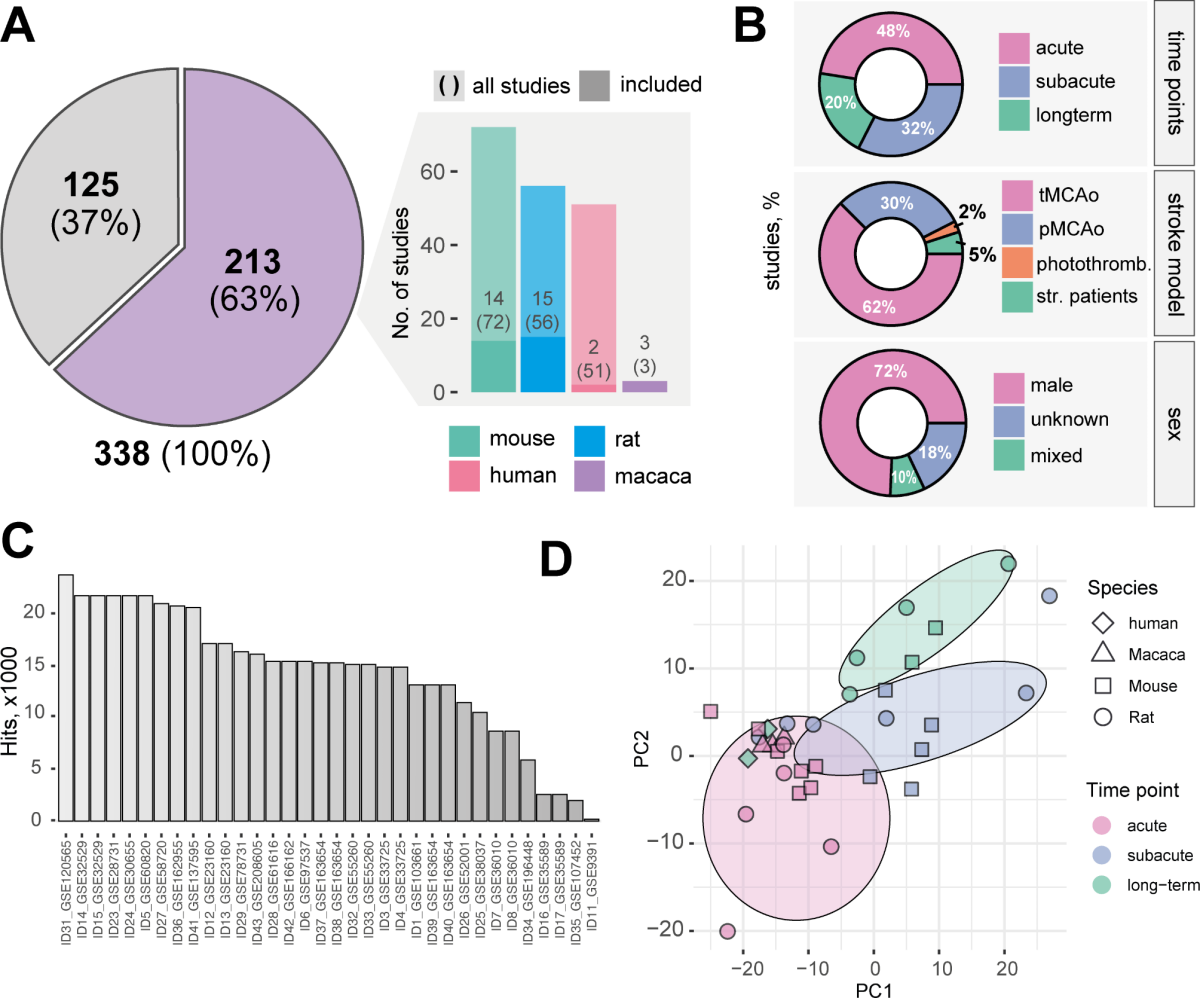
Overview of studies dissecting stroke-related gene expression differences in the brain. (**A**) (left) Excluded and included stroke studies from NCBI GEO. (Right) Exclusion of datasets subdivided by species (mouse, rat, human and primates) with missing information, duplicates, pooled samples, poor quality controls, and no clear separation between stroked and non-stroked groups were further excluded. (**B**) Distribution of individual studies by time point, stroke model and sex. (**C**) Number of gene hits in individual studies. More information about the IDs can be found in Suppl Table 1. (**D**) Principal component analysis (PCA) of all stroke studies across different species and time points

In total, 338 studies were screened that examined gene-expression differences in stroke. Of these, 213 studies analyzed brain tissue and used non-stroked brain tissue controls. Datasets with missing information, duplicates, pooled samples, poor quality controls, and no clear separation between stroked and non-stroked groups were further excluded. In total, we included 34 datasets from mice (12), rats (15), humans (2) and primates (3) (Fig. 1A). The datasets included in this meta-analysis compared gene expression in stroked brain tissue to control brain tissue (either from intact, sham operated or contralesional brain regions). Detailed information of each dataset is provided in Suppl. Table 1 describing the GEO ID, sample type, control type, stroke model, time point, sex, and further information.

The majority of datasets were acquired either in the acute, (< 24 h: 49%) or subacute (> 24 h and < 7d: 36%) phase after stroke, only few datasets investigated long-term gene expression changes after stroke (> 7d: 10%). The most frequently applied model of stroke was the tMCAo (67%), followed by the pMCAo model (31%) only 2% of the studies used the photothrombotic stroke model. Surprisingly, the vast majority of datasets only used the male sex (74%) (Fig. 1B). The majority of datasets had data from 10 to 25k genes, only four micro-array studies showed data for less than 10k hits (all of them were derived from human and primate samples) (Fig. 1C). Principal component analysis (PCA) revealed the widest separation across the datasets for the acute, subacute, and long-term periods after stroke indicating the importance of time in the transcriptomic signatures (Fig. 1D).

### Gene expression differences in stroked brain tissue between mice and rats are pronounced in acute phase after injury

The vast majority of stroke studies are performed in either rats or mice, hence the focus of the subsequent analysis was on the more detailed rodent datasets.

**Fig. 2:**
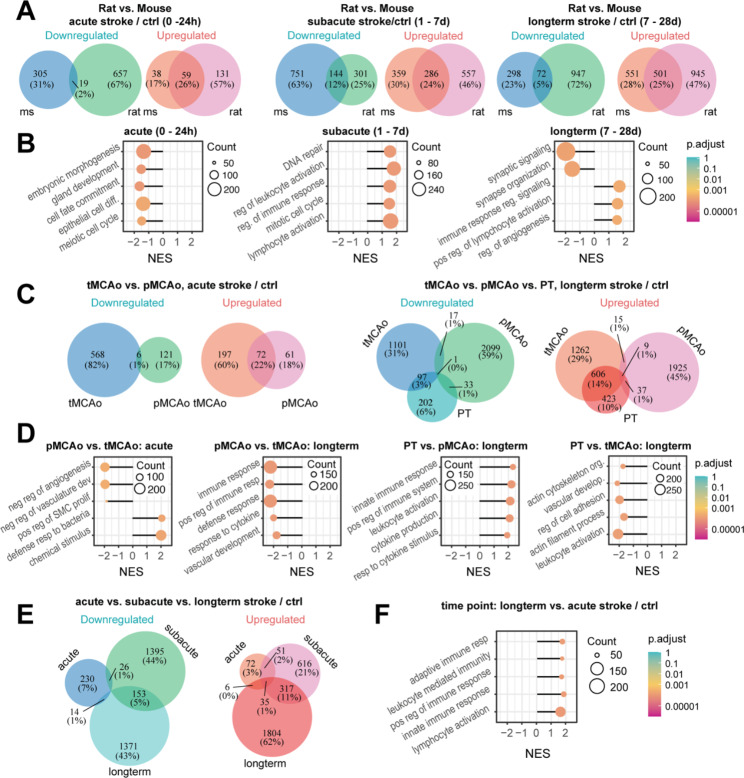
Gene expression profiles after stroke differ across species. (**A**) Venn Diagram representing number of shared and differentially expressed genes between mice and rats to different time points. (**B**) Gene ontology analysis of a subset of most significantly enriched biological processes in rats and mice. (**C**) Venn Diagram representing number of shared and differentially expressed genes between permanent MCAo and transient MCAo and the photothrombotic stroke model at different time points. (**D**) Gene ontology analysis of a subset of most significantly enriched biological processes in different stroke models. (**E**) Venn Diagram representing number of shared and differentially expressed genes between different time periods after stroke. (**F**) Gene ontology analysis of a subset of most significantly enriched biological processes at different time points

To analyze the effect of the species, the transcriptomes were compared at the acute, subacute, and long-term timepoints after MCAo stroke induction. Analysis of differentially expressed genes (DEGs) revealed that there was a greater overlap between upregulated genes (~ 25%) in the mouse and rat transcriptome to all time points (Fig. 2A, Suppl. Figure 1). Most upregulated genes acutely after stroke were as expected early-response and inflammation associated genes such as *Hspa1a, Cxcl1, Ccl3, and Fos*. For downregulated genes the overlap was higher at the subacute and long-term time point (shared downregulated genes: acute: 1%, subacute: 12%, long-term: 5%, Fig. 2A). A list of the 60 most DEG can be found in Suppl. Tables 2–4.

Gene ontology (GO) analysis was carried out to identify the biological function of the DEGs in the mouse and rat stroke transcriptome. Interestingly, most significantly enriched GO terms were distinct at the different time points. While only 10% of the top30 GO terms in the acute phase were stroke-related, the majority of enriched GO terms in the subacute and long-term time point were directly related to the stroke pathology. For instance, inflammation related GO terms (e.g., immune system response, regulation of leukocyte activation) were differentially enriched in the subacute and long-term phase between mouse and rat. In the long-term, differences in the synaptic signaling and synaptic organization were among the top enriched GO terms (Fig. 2B, Suppl. Tables 8–10).

### Different stroke-models have distinct gene expression profiles in stroked brain tissue

Acutely after stroke transient MCAo (tMCAo) and permanent MCAo (pMCAo) are the most suitable models to mimic acute human stroke cascade [7]. These methods enable either permanent occlusion of the blood vessels or transient ischemia with reperfusion.

First gene expression datasets from stroked tissue were compared in rodents using tMCAo to pMCAo procedure at the acute (< 24 h) time period. The total number of up- and downregulated DEG was higher following tMCAo acutely after stroke. While 22% of common genes were upregulated in both models only 1% of common genes were downregulated acutely after stroke indicating a unique stroke signature between tMCAo and pMCAo models (Fig. 2C, Suppl. Table 5). Most significantly enriched pathways in pMCAo included detection of chemical stimulus and innate defense responses whereas most enriched pathway in tMCAo included e.g. regulators of vascular and smooth muscle cell responses (Fig. 2D, Suppl. Table 11).

Apart from MCAo models, long-term recovery after stroke can additionally be evaluated using a photothrombotic stroke model (PT). Although the PT model does cause a vasogenic edema acutely after stroke (that is uncharacteristic for human stroke), the method is minimally invasive and results in well-characterized long-term sensory-motor deficits and gradual incomplete recovery [10, 15]. DEG were compared in stroked tissue of pMCAo, tMCAo and PT models at long-term (7–28 d) period. More genes were differentially expressed in pMCAo and tMCAo at a long-term period compared to the acute phase after stroke. A higher overlap could be observed for downregulated and upregulated genes (18%) in tMCAo and PT model, whereas long-term pMCAo gene expression had a highly unique molecular signature compared to the other stroke models (tMCAo: 3%; PT: 3%) (Fig. 2C, Suppl. Table 6). Most significantly enriched pathways in the tMCAo model compared to pMCAo and PT were related to regulators of immune responses e.g., positive regulation of immune system responses, regulation of cytokine production, leukocyte activation indicating a considerably stronger immune activity in tMCAo model. Apart from immune system related pathways, the PT stroke model showed reduced enrichment in remodeling related pathways including, regulation of cell adhesion, actin cytoskeleton organization and tissue and blood vessel morphogenesis compared to the tMCAo model (Fig. 2D Suppl. Tables 12–14).

### Stroke gene expression signature is unique in the acute, subacute, and long-term post-injury time periods

As the highest deviation in the transcriptome signature after stroke appears to be dependent on the time point after stroke (Figs. 1D and 2E, Suppl. Table 7), I investigated which pathways were enriched to the different time points.

Most (adaptive) immune-related pathways were strongly upregulated at the long-term time point compared to the acute phase after stroke such as lymphocyte activation, positive regulation to immune response, and adaptive immune response (Fig. 2F, Suppl. Figure 2). However, in the subacute phase many processes involving synapse activation and signaling as well as neurotransmitter secretion were downregulated compared to the acute phase. Interestingly, these pathways e.g., synaptic signaling and synaptic plasticity appear to be re-activated at the long-term phase after stroke potentially indicating endogenous repair and remodeling processes (Fig. 2F, Suppl. Figure 2, Suppl Tables 15–17).

## Discussion

Dissecting the molecular profile after stroke promises to identify new targets for potential therapeutic compounds. However, the multifaceted ischemic cascade and the variety of used animal models complicates the search of promising pathways related to stroke. Here, major differences were identified in publicly available gene expression profiles after stroke that varied depending on the time period, the animal/rodent species, and the stroke model used. These alterations affected primarily upregulated genes and affected both general and stroke-related pathways. The biggest alteration in the gene expression was identified for the different time periods after stroke, supporting the hypothesis that timing of therapy in stroke is of primary importance [16].

Furthermore, RNA datasets from primates and humans were only comparable to a limited extent with the rodent datasets. The human and primate datasets had considerably fewer analysed genes as they were derived from micro-array studies. Additionally, obtaining high quality RNA from human stroked brain tissue is challenging, mainly because of the time gap between the patient’s death and the collection of tissue, which can contribute to the decline in RNA quality.

The heterogenicity of the stroke pathophysiology is a further limitation in identifying novel targets for therapy. For instance, the molecular signature of the stroke core may considerably vary from the penumbra and different cell types may contribute to gene expression changes. However, there are only a limited number of studies that have thoroughly investigated these differences [17]. Novel advancements with single cell/nucleus and spatially resolved transcriptomics may provide further insights in near future [18, 19].

Many detrimental and regenerative processes after stroke occur in parallel over time. The classification of time points after stroke in acute (0-24 h), subacute (1-7d) and long-term (7-28d) was based on previous preclinical studies and studies used in this meta-analysis. The general aim in this classification was to describe gene expression differences in time frames often used to evaluate treatments that either inhibit with the initial stroke progression (acute), reduce inflammatory responses or are neuroprotective (subacute) and regenerative treatments (long-term). For instance, interference with early-response pathways such as CCL2 are often investigated acutely after stroke within the first 24 h [20]. Anti-inflammatory drugs blocking with subacute post-ischemic inflammation are usually tested in the first 24-7d after stroke [21] and therapies aimed to improve long-term recovery evaluate the effects later than 7d [9, 10, 22]. A limitation of the study is that dynamic changes in gene expression in between these time periods are not detectable. A potential alternative could involve employing a data-driven unsupervised approach to establish time frames that are not reliant on previous research, but rather on gene network behavior. This method could enable future investigators to design interventions more effectively.

Species-dependent differences may be attributed to differences in the brain circulation of mice and rats. While the overall function and structure of the brain vasculature are similar in both mice and rats, the Circle of Willis is more developed in rats than that in some strains of mice including C57Bl6 [23, 24]. These differences may affect the collateral blood perfusion and are known to considerably vary even within mice of different genetic backgrounds [24, 25].

Many differentially expressed genes were also associated with inflammatory responses after stroke. It has previously been shown that mice and rats exhibit distinct inflammatory reactions after spinal cord injury [26]. These differences include for instance timing and magnitude of immune cell infiltration [26], also more recent studies indicate sex-specific differences within the species that could not be assessed in this analysis [27].

This meta-analysis reveals that considerable gene expression differences exist between mice and rats as well as the used stroke models. This data supports the recommendations to confirm the effect of experimental therapeutic compounds in at least two animal species or different stroke models [28]. Additionally, the study was designed to examine the effect of sex on gene expression after stroke. However, there is still a lack of datasets for the female sex after stroke as all studies used male rodents or mixed sex. The sex plays an important role in human stroke pathology [29] and should be further investigated in preclinical gene expression studies in near future.

## Conclusion

In sum, this meta-analysis identifies distinct gene expression changes in stroked brain tissue across species, time points and stroke models. These differences affected general and stroke-related pathways and need to be considered when evaluating new potential therapeutic compounds.

## Electronic supplementary material

Below is the link to the electronic supplementary material.

Supplementary Material 1

Supplementary Material 2

## Data Availability

All raw data are available in the supplementary files of the manuscript.
